# Effects of Fermented Longan Peel (*Dimocarpus longan*) on Growth Performance, Digestive Enzyme Activity, Intestinal Microstructure, Immune Response, and Gene Expression of Nile Tilapia (*Oreochromis niloticus*) Raised Under Biofloc System

**DOI:** 10.3390/antiox15030394

**Published:** 2026-03-20

**Authors:** Supreya Wannavijit, Punika Ninyamasiri, Wanarsa Nonkrathok, Sudaporn Tongsiri, Phisit Seesuriyachan, Yuthana Phimolsiripol, Seyed Hossein Hoseinifar, Hien Van Doan, Marina Paolucci

**Affiliations:** 1 Department of Animal and Aquatic Sciences, Faculty of Agriculture, Chiang Mai University, Chiang Mai 50200, Thailand; supreya_wan@cmu.ac.th (S.W.); punika_n@cmu.ac.th (P.N.); 2 Faculty of Fisheries Technology and Aquatic Resources, Maejo University, Chiang Mai 50290, Thailand; mju6510301004@mju.ac.th (W.N.); sudaporn@mju.ac.th (S.T.); 3 Thailand Faculty of Agro-Industry, Chiang Mai University, Chiang Mai 50100, Thailand; phisit.s@cmu.ac.th (P.S.); yuthana.p@cmu.ac.th (Y.P.); 4 Department of Fisheries, Faculty of Fisheries and Environmental Sciences, Gorgan University of Agricultural Sciences and Natural Resources, Gorgan 4913815739, Iran; hoseinifar@alumni.ut.ac.ir; 5 Functional Feed Innovation Center (FuncFeed), Faculty of Agriculture, Chiang Mai University, Chiang Mai 50200, Thailand; 6 Department of Sciences and Technologies, University of Sannio, 82100 Benevento, Italy

**Keywords:** fermented longan peel, Nile tilapia, digestive enzymes, immune response, biofloc

## Abstract

The valorization of agricultural by-products as functional feed additives represents a promising strategy for sustainable aquaculture. This study evaluated the effects of dietary fermented longan peel (FLP), produced through enzymatic hydrolysis and *Lactiplantibacillus plantarum* fermentation, on growth performance, digestive physiology, gut morphology, innate immunity, and gene expression in Nile tilapia (*Oreochromis niloticus*) cultured under a biofloc system. Five experimental diets were formulated with graded FLP levels (0, 5, 10, 20, and 40 g kg^−1^) and fed to fish for eight weeks. Growth indices, including final weight, weight gain, and specific growth rate, improved significantly in fish receiving 20 g kg^−1^ FLP, following a strong quadratic response pattern. *In vitro* digestibility assays showed enhanced carbohydrate and protein digestibility, coinciding with increased intestinal amylase and protease activities. Histological analysis indicated that moderate FLP inclusion (10–20 g kg^−1^) promoted villus height, crypt depth, and epithelial organization. Innate immune parameters, including lysozyme, peroxidase, and alternative complement activity, were markedly elevated in serum and mucus, particularly at 20–40 g kg^−1^ after eight weeks. Gene expression profiling revealed significant up-regulation of growth-related (*IGF-1*, *GH*, *NPY-α*, *Galanin*), immune-related (*TLR-7*, *TNF-α*, *NFκB*), and antioxidant-related (*hsp70*, *Keap-1*, *nrf-2*, *GST-α*) genes in fish fed higher FLP levels, with responses plateauing beyond 20 g kg^−1^. Overall, FLP supplementation at 20 g kg^−1^ optimally enhanced growth, digestive efficiency, intestinal health, and innate immune status. These findings demonstrate the potential of fermented longan peel as a cost-effective, bioactive, and sustainable functional feed ingredient for tilapia and other warm-water aquaculture species.

## 1. Introduction

Aquaculture is the fastest-growing food production sector worldwide and plays a crucial role in ensuring global food security, nutrition, and rural employment [1]. It currently supplies more than half of the fish consumed by humans and is projected to expand further as capture fisheries reach their limits [2]. However, intensive aquaculture production faces significant challenges, including the high cost of conventional feed ingredients, dependence on fishmeal and fish oil, and the increasing occurrence of disease outbreaks [3]. These issues underscore the need for sustainable feed alternatives that can improve growth, immunity, and environmental performance while supporting a circular bioeconomy [4]. Among cultured species, Nile tilapia (*Oreochromis niloticus*) is one of the most economically important freshwater fish due to its rapid growth, omnivorous feeding behavior, and tolerance to various farming conditions [5]. It is widely cultivated in Asia, Africa, and Latin America and represents a major protein source for human consumption [6]. Nonetheless, intensive rearing systems often expose tilapia to environmental stress, poor water quality, and pathogenic infections, leading to impaired immunity and reduced productivity [5,7]. In this context, the use of natural functional feed additives derived from agricultural by-products has been recognized as a promising strategy to enhance fish health and sustainability in tilapia farming.

Longan (*Dimocarpus longan* Lour.) is a tropical fruit widely grown in Thailand, Vietnam, and China [8], with annual global production approximately of 3.44 million tons [9,10]. Longan peel accounts for 12.4–19.6% of the whole fruit weight [11]. During longan pulp processing, tens of thousands of tons of peel and seeds are discarded annually, causing environmental pollution and resource wastage. Thus, the reuse of longan peel and seeds has significant development potential, but there is a lack of a systematic review of the active ingredients, health benefits and applications of longan plants [8]. Studies indicate that the longan peel and seeds are rich in polyphenols, flavonoids, and polysaccharides with antioxidant, antityrosinase, antibacterial, antifungal, antidiabetic, and other activities [12,13]. The longan peel phenolic compounds include ellagic acid, gallic acid and corilagin in free, esterified- and etherified forms [14]. Recent studies demonstrated that longan peel powder improved growth performance, immune activity, and gene expression in Nile tilapia, suggesting its potential as a functional feed additive [15]. Nevertheless, its high fiber and antinutrient contents may limit digestibility and nutrient absorption [8,14]. Fermentation represents an efficient bioprocessing approach to enhance the nutritional and functional value of plant-based ingredients [16,17]. Through microbial metabolism, fermentation degrades complex carbohydrates and antinutritional factors, increases bioactive compound availability, and produces beneficial metabolites such as organic acids and enzymes [18,19]. Fermented plant materials have been shown to improve feed utilization, gut health, and immune status in aquaculture species [20,21,22,23]. In particular, fermentation with lactic acid bacteria like *Lactobacillus* enhances antioxidant potential and contributes to the production of probiotics that positively influence the intestinal microbiota of fish [24,25].

In addition to functional feeds, the biofloc technology (BFT) system has gained prominence as an environmentally friendly aquaculture practice that promotes nutrient recycling, microbial biomass formation, and disease resistance [26]. BFT supports beneficial microbial consortia that improve digestion, water quality, and immunity in cultured fish, making it an excellent platform for testing functional additives [27]. Integrating fermented agro-industrial by-products with biofloc systems can provide synergistic benefits, enhancing nutrient bioavailability, stimulating immune responses, and reducing production costs.

Recent studies suggest that combining fermented agricultural or industrial by-products with biofloc systems may produce additive or synergistic benefits [28,29]. Fermented plant substrates introduced into biofloc environments can serve as additional carbon sources, modulate microbial community structure, and enhance microbial protein production [27,30]. Moreover, fermentation-derived bioactive may interact with biofloc microbial consortia to increase short-chain fatty acid production, improve intestinal epithelial integrity, and stimulate immune signaling pathways [31]. Emerging evidence indicates that such integrated strategies can enhance nutrient bioavailability, digestive enzyme activity, antioxidant capacity, and non-specific immune responses in cultured fish species [25,32,33,34]. These findings support the concept that dietary fermentation and microbial-based rearing systems should not be considered independent interventions but rather interconnected components of a functional aquaculture microbiome. Despite increasing evidence on the benefits of both longan peel and fermented plant materials in aquaculture, the effects of fermented longan peel supplementation in Nile tilapia cultured under biofloc systems remain unexplored. Understanding how fermentation-derived metabolites and biofloc microorganisms interact to influence fish physiology is essential for optimizing feed strategies that align with sustainable production goals. Therefore, this study aimed to evaluate the effects of dietary fermented longan peel (FLP) supplementation on growth performance, digestive enzyme activity, intestinal microstructure, immune response, and expression of growth-, immune-, and antioxidant-related genes in Nile tilapia reared under a biofloc system. The findings are expected to advance knowledge on the functional application of fruit by-products in aquafeeds and contribute to sustainable aquaculture practices through the combined use of fermentation and biofloc technology.

## 2. Materials and Methods

### 2.1. Production of Fermented Longa Peel

#### 2.1.1. Sample Preparation

Longan peel material was first collected and thoroughly washed to remove impurities. The material was then dehydrated in a hot-air oven at 50 °C for 48 h to remove moisture. After drying, the peels were ground into a fine powder and passed through a 100-mesh sieve to ensure uniform particle size. The resulting powder was subsequently stored in sealed plastic bags at 4 °C until further processing.

#### 2.1.2. Pretreatment Process

Longan peel powder (10 g) was mixed with 0.5% sodium hydroxide solution and subjected to high-pressure treatment at 50 MPa for either 5 or 20 min. After pressurization, the material was thoroughly rinsed with deionized water to remove residual alkali. The treated samples were then dried in a hot-air oven at 80 °C for 24 h and subsequently stored at 4 °C until further analysis, following the general procedure described by Sahare, Singh, Laxman and Rao [35].

#### 2.1.3. Enzymatic Hydrolysis

Pressure-treated longan peel (4 g) was suspended in 40 mL of 0.1 M citrate buffer (pH 4.8) and subjected to enzymatic digestion using iKnowZyme PXC (REACH BIOTECHNOLOGY Co., Ltd., Thanyaburi, Thailand) at a dosage of 100 µg per gram of substrate. The mixture was incubated at 55 °C with continued shaking at 150 rpm for 72 h. After hydrolysis, the suspension was centrifuged at 12,000× *g* for 10 min, and the resulting supernatant was collected for determination of reducing sugar content and antioxidant properties [36].

#### 2.1.4. Preparation of Starter Cultures for Fermentation

The bacterial starter culture was prepared using *Lactiplantibacillus plantarum* TISTR 2265, obtained from the Thailand Institute of Scientific and Technological Research. This strain has been reported to process significant biotechnological potential, including rapid growth and the capacity to enhance GABA production, total phenolic content, and antioxidant capacity [37]. The strain was initially cultured in MRS broth at 37 °C for 24 h. Subsequently, 5% (*v*/*v*) of the activated culture was transferred into fresh MRS broth and incubated under the same condition until optical density at 600 nm reached 0.6–0.8. The bacterial cells were then harvested by centrifugation at 6000× *g* for 15 min at 4 °C, washed twice with 0.9% NaCl, and finally adjusted to a cell density of approximately 7–8 log CFU mL^−1^.

#### 2.1.5. Production of Fermented Longan Peel

Following the 72 h enzymatic hydrolysis, the longan peel mixtures were sterilized at 121 °C for 15 min. After cooling to room temperature, the substrates were aseptically inoculated with 5% (*w*/*v*) of the prepared *L. plantarum* TISTR 2265 culture. Fermentation was conducted at 30 °C for 72 h under controlled conditions. After fermentation, the mixtures were centrifuged at 6000 rpm for 15 min, and the resulting solid fraction was collected for further analyses.

The proximate composition and bioactive compound profile of the fermented longan peel (FLP) are presented in Table 1 and Table 2. Moisture, dry matter, ash, crude protein, and lipid contents were determined according to AOAC procedures [38]. Quantification of major phenolic compounds was conducted following the methods described by Dhanani, Shah and Kumar [39] and Pereira, Câmara, Cacho and Marques [40]. Antioxidant properties, including ABTS^+^ scavenging ability, FRAP, total flavonoids, DPPH (IC_50_), and total phenolics, was evaluated using established protocols [41,42].

### 2.2. Experiment Diets

A basal diet was formulated according to the established nutrient requirements of Nile tilapia, following the guidelines described by Wannavijit, Outama, Le Xuan, Lumsangkul, Lengkidworraphiphat, Tongsiri, Chitmanat and Doan [43] (Table 3). Experimental diets were prepared by supplementing the basal formulation with graded levels of fermented longan peel (FLP). All dry ingredients were finely ground, accurately weighed, and thoroughly mixed using a mechanical mixer to ensure homogeneity. Subsequently, fish oil and soybean oil were gradually incorporated to achieve uniform distribution within the feed matrix. Distilled water (approximately 300 mL per kg of diet) was then slowly added to obtain an appropriate consistency for pellet formation. The resulting dough was processed through a pelletizer (Siam Farm Services Co., Ltd., Lampang 52130, Thailand) to produce uniform pellets. The pellets were subsequently dried and stored under appropriate conditions until use.

### 2.3. Experiment Design

Male Nile tilapia (*Oreochromis niloticus*) fingerlings were obtained from PC Farm, Thailand. Upon arrival, the fish were acclimated for 30 days and fed a commercial diet (CP 9950,). This was followed by a two-week adaptation period during which the fish were fed the control experimental diet. After acclimation, a total of 300 fish with an initial weight of 15.04 ± 0.03 g were randomly distributed into 15 aerated tanks (150 L each), with 20 fish per tank. The experiment was conducted using a Completely Randomized Design (CRD) consisting of five dietary treatments, each with three replicates. Fish were fed their respective experimental diets to apparent satiation twice daily at 08:30 and 16:30 for eight weeks.

### 2.4. Biofloc Water Preparation and Management

Two weeks prior to fish stocking, biofloc formation was initiated in each tank by adding 2 g of fish feed, 400 g of salt, 5 g of dolomite, and 5 g of molasses as a carbon source to stimulate heterotrophic bacterial proliferation. Tanks were continuously aerated to maintain suspended microbial aggregates and prevent sedimentation. Visible floc formation and increased water turbidity were observed within the conditioning period, indicating successful establishment of microbial aggregates before the start of the feeding trial.

Throughout the experiment, the carbon-to-nitrogen (C:N) ratio was maintained at 15:1 by daily supplementation of molasses (40% carbon), following the protocol of Avnimelech [44]. The C:N ratio was calculated based on dietary nitrogen input and estimated residual nitrogen in each tank, as described by Cardona, Lorgeoux, Chim, Goguenheim, Le Delliou and Cahu [45]. Water quality parameters, including ammonium, pH, dissolved oxygen, and floc volume were monitored regularly and remained within acceptable ranges (NH_3_ = 0.19 ± 0.01 mg L^−1^, pH = 7.86 ± 0.68, dissolved oxygen = 5.73 ± 0.48 mg L^−1^, and floc volume = 8.97 ± 0.46 mL L^−1^) for tilapia culture throughout the experimental period.

### 2.5. Growth Performance Measurements

Growth performance and survival of Nile tilapia were evaluated using standard aquaculture performance indicators. The following parameters were calculated: Weight gain (WG) = final weight (g) − initial weight (g); Specific growth rate (SGR %) = 100 × (ln final weight − ln initial weight)/total duration of experiment; Feed conversion ratio (FCR) = feed given (dried weight)/weight gain (wet weight), and Survival rate (%) = (final fish number/initial fish number) × 100.

### 2.6. In Vitro Digestibility and Digestive Enzyme Activity

#### 2.6.1. Crude Enzyme Extraction

Crude enzyme extracts were prepared from small intestinal tissue by homogenizing samples in 0.2 M phosphate buffer (pH 8.0) at a 1:3 (*w*/*v*) tissue-to-buffer ratio, following the method described by Rungruangsak-Torrissen [46]. The homogenate was then centrifuged at 15,000× *g* for 15 min at 4 °C, and the resulting supernatant was carefully collected while avoiding the upper lipid layer. The collected supernatant served as the crude enzyme extract. All samples were subsequently stored at −80 °C until further analysis [47].

#### 2.6.2. Digestive Enzyme Assays

Amylase activity was determined according to the method described by Areekijseree, Engkagul, Kovitvadhi, Thongpan, Mingmuang, Pakkong and Rungruangsak-Torrissen [48], using 5% soluble starch as the substrate. The absorbance was measured at 540 nm, and enzyme activity was calculated based on a maltose standard curve, expressed as mg maltose h^−1^ mg^−1^ protein. Lipase activity was measured following the procedure of Rungruangsak-Torrissen [46], using 0.01 M p-nitrophenyl palmitate as the substrate. The absorbance was recorded at 410 nm, and activity was quantified using a p-nitrophenol standard curve, expressed as µmol p-nitrophenol h^−1^ mg^−1^ protein. Total protease activity was assayed using 5% azocasein according to Areekijseree, Engkagul, Kovitvadhi, Thongpan, Mingmuang, Pakkong and Rungruangsak-Torrissen [48]. One unit of enzyme activity (U) was defined as the amount of enzyme producing a 1.0-unit increase in absorbance at 440 nm under assay conditions, and the activity was expressed as U h^−1^ mg^−1^ protein. Trypsin activity was determined using 1.25 mM benzoyl-DL-arginine-p-nitroanilide as the substrate [46]. The absorbance was measured at 410 nm, and the enzyme activity was calculated using a p-nitroanilide calibration curve, expressed as µmol p-nitroaniline h^−1^ mg^−1^ protein.

#### 2.6.3. *In Vitro* Digestibility

Crude enzyme extracts were dialyzed overnight in 50 mM phosphate buffer (pH 8.0) prior to use in the *in vitro* digestibility assay. Experimental diets were finely ground to serve as substrates. Protein and carbohydrate digestibility were evaluated using crude intestinal enzymes from fish, following a modified protocol of Rungruangsak-Torrissen, Rustad, Sunde, Eiane, Jensen, Opstvedt, Nygård, Samuelsen, Mundheim and Luzzana [49].

For each assay, 5 mg of dried diet powder was mixed with 10 mL of 50 mM phosphate buffer (pH 8.0), 50 μL of 0.5% chloramphenicol, and 125 μL of dialyzed crude enzyme extract. The mixture was incubated at 25 °C in a shaking incubator for 24 h to mimic digestion in tropical fish. A baseline control (T0) was obtained by withdrawing 0.5 mL of the reaction mixture before enzyme addition, immediately heating it at 100 °C for 5 min to stop all enzymatic activity, and storing it at −80 °C for subsequent analysis. Digestion was initiated by adding 0.5 mL of dialyzed enzyme extract with standardized trypsin activity. After 24 h of incubation, 1 mL of the digested suspension was collected, heat-inactivated (100 °C, 5 min), and preserved at −80 °C for further measurements.

Protein digestibility was quantified using the trinitrobenzene sulphonic acid (TNBS) method, which detects free amino groups released during hydrolysis. A 200 μL aliquot of the digested sample was mixed with 2 mL of phosphate buffer (50 mM, pH 8.0) and 1 mL of 0.1% TNBS, followed by incubation in the dark at 60 °C for 1 h. The reaction was stopped with 1 mL of 1 M HCl, and absorbance was measured at 420 nm. Values were calculated using a DL-alanine standard curve and expressed as µmol DL-alanine per g of feed per unit of trypsin activity, correcting for enzyme variability [50].

Carbohydrate digestibility was determined by measuring reducing sugars using the dinitrosalicylic acid (DNS) assay. One milliliter of digested sample was reacted with 500 μL of DNS reagent, boiled for 5 min, cooled, and read at 540 nm. Reducing sugar concentrations were derived from a maltose standard curve and expressed as mg maltose per g of feed per unit of amylase activity, enabling comparisons across treatments [50].

### 2.7. Intestinal Microstructure

During sampling, mid-intestinal segments were collected from six fish in each treatment group (2 fish per tank). Tissues were gently rinsed with phosphate-buffered saline (PBS; pH 7.4) and fixed in 10% neutral-buffered formalin for 24 h. After fixation, samples were processed for routine histology: they were dehydrated, embedded in paraffin, sectioned at 4 μm thickness, and stained with hematoxylin and eosin (H&E) following the procedure outlined by Ruiz, Owatari, Yamashita, Ferrarezi, Garcia, Cardoso, Martins and Mouriño [51]. Prepared slides (3 replication per sample) were examined under a compound microscope (Leica DM750, Leica Microsystems, Wetzlar, Germany) at 20× magnification. Morphometric parameters, including villus height (VH), villus width (VW), and crypt depth (CD), were quantified using Leica LAS X imaging software version 3.9.0 (Leica Microsystems, Wetzlar, Germany).

### 2.8. Evaluation of Innate Immune Responses

#### 2.8.1. Sample Preparation

Fish samples (three per tank) were collected at weeks 4 and 8 for analyses of skin mucus and serum immune parameters.

Skin mucus collection followed a modified version of Quade and Roth [52] as adapted by Van Doan, Hoseinifar, Naraballobh, Paolucci, Wongmaneeprateep, Charoenwattanasak, Dawood and Abdel-Tawwab [53]. Fish were anaesthetized with clove oil (5 mL L^−1^) and placed in polyethylene bags containing 10 mL of 50 mM NaCl. Gentle downward strokes along the body surface for approximately one minute facilitated mucus release. The solution was transferred to 15 mL tubes and centrifuged at 1500× *g* for 10 min at 4 °C. About 1 mL of the supernatant was aliquoted into Eppendorf tubes and stored at −80 °C until analysis.

Serum samples were obtained following Van Doan, Wannavijit, Tayyamath, Quynh, Sumon, Linh, Seesuriyachan, Phimolsiripol, Esteban and Gisbert [25]. Blood was drawn from the caudal vein using 1 mL syringes, left to clot at room temperature for 1 h, and then refrigerated at 4 °C for several hours. Serum was separated and stored at −80 °C for later assays.

#### 2.8.2. Lysozyme Activity

Lysozyme activity in both skin mucus and serum (3 replications per fish) was quantified following the method of Parry Jr, Chandan and Shahani [54], with modifications described by Wannavijit, Outama, Le Xuan, Lumsangkul, Lengkidworraphiphat, Tongsiri, Chitmanat and Doan [43].

#### 2.8.3. Peroxidase Activity

Peroxidase activity (3 replications per fish) was quantified following Quade and Roth [52] with slight adjustments from Wannavijit, Outama, Le Xuan, Lumsangkul, Lengkidworraphiphat, Tongsiri, Chitmanat and Doan [43].

#### 2.8.4. Alternative Complement Pathway Activity (ACH50)

ACH50 activity (2 replications per fish) was measured using a modified hemolytic assay based on Yano, Ando and Nakao [55] with some adjustments as described in [25].

### 2.9. Genes Expression Analysis

Relative expression levels of immune- and antioxidant-related genes were evaluated using two fish from each tank. Fish were anesthetized with clove oil (100 mg mL^−1^), after which the liver, hindgut, and head kidney were dissected and immediately processed for RNA extraction. Total RNA was isolated using either TRIzol Reagent (Life Technologies, Carlsbad, CA, USA) or the PureLink™ RNA Mini Kit (Invitrogen, Carlsbad, CA, USA). RNA concentration and purity were assessed with a NanoDrop™ One spectrophotometer (Thermo Fisher Scientific, Waltham, MA, USA) by measuring absorbance at 260 and 280 nm.

A total of 1000 ng RNA from each sample was reverse-transcribed into cDNA using the iScript™ cDNA Synthesis Kit (BIO-RAD, Laboratories, Hercules, CA, USA). The primers used in this study were adopted from previously published studies and have been validated for gene expression analysis in Nile tilapia (Table 4). Quantitative real-time PCR (qRT-PCR) was performed in triplicate using 100 ng cDNA, 400 µM of each primer, and iTaq Universal SYBR Green Supermix (2×) on a CFX96 Touch Deep Well Real-Time PCR System (BIO-RAD, USA). The PCR cycling parameters followed Le Xuan, Vu Linh, Wannavijit, Outama, Lubis, Machimbirike, Chromkaew, Phimolsiripol and Van Doan [56]; an initial denaturation at 95 °C for 30 s, followed by 40 cycles at 95 °C for 15 s and 60 °C for 30 s, and a melt-curve stage consisting of 95 °C for 15 s, 60 °C for 60 s, and 95 °C for 15 s. Relative gene expression was calculated using the 2^−ΔΔCt^ method Livak and Schmittgen [57], supported by standard-curve validation for amplification efficiency. by, with standard curve analysis.

### 2.10. Statistical Analysis

Data normality was examined using the Kolmogorov–Smirnov test. One-way ANOVA was performed to assess treatment effects, and significant differences among means were identified using Duncan’s multiple range test. All analyses were conducted using GraphPad Prism version 10 (GraphPad Software, San Diego, CA, USA). Statistical significance was set at *p* < 0.05.

**Figure 1:**
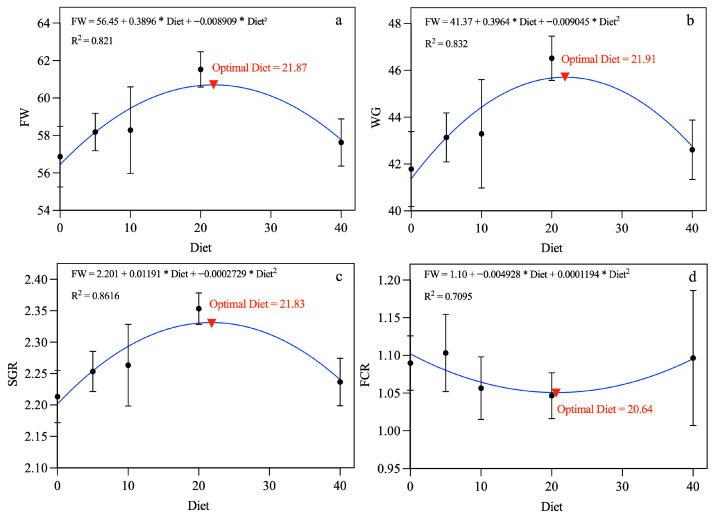
Growth performance and feed utilization of Nile tilapia fed with different dietary levels of fermented longan peel. Polynomial regression curves show the relationship between diet and (**a**) final body weight, (**b**) weight gain, (**c**) specific growth rate, and (**d**) feed conversion ratio. Optimal dietary levels are indicated by red arrows.

The optimal dietary inclusion level of fermented longan peel (FLP) was estimated using quadratic regression analysis based on growth performance indicators, including final weight (FW), weight gain (WG), specific growth rate (SGR), and feed conversion ratio (FCR), as shown in Figure 1. The quadratic model used in the analysis was: Y = a + bX + cX^2. Here, Y represents the response variable (FW, WG, SGR, or FCR), X represents the dietary FLP inclusion level, and a, b, and c are regression coefficients estimated using GraphPad Prism version 10 (GraphPad Software, San Diego, CA, USA).

The optimal inclusion level was calculated from the vertex of the quadratic equation using the formula: X_opt = −b/2c, where X_opt represents the estimated optimal dietary level. Based on this analysis, the optimal FLP inclusion levels were estimated to be 21.87 g kg^−1^ for FW, 21.91 g kg^−1^ for WG, 21.83 g kg^−1^ for SGR, and 20.64 g kg^−1^ for FCR, as illustrated in Figure 1.

In addition, prior to statistical analysis, data normality was tested using the Kolmogorov–Smirnov test. Differences among treatments were evaluated using one-way ANOVA, followed by Duncan’s multiple range test for mean comparisons. All analyses were performed using GraphPad Prism version 10, and statistical significance was considered at *p* < 0.05.

## 3. Results

### 3.1. Growth Parameters

The growth performance and feed utilization indices of Nile tilapia (*O. niloticus*) fed diets containing graded levels of fermented longan peel (FLP) for eight weeks are summarized in Table 5 and Figure 1. Initial body weights (IW) were comparable among all groups, ranging from 15.00 to 15.08 g, indicating uniformity at the start of the trial.

After eight weeks, final body weight (FW) increased progressively with higher dietary FLP inclusion, with the FLP20 group achieving the greatest weight (61.53 ± 0.55 g), compared with the control group (56.87 ± 0.94 g). This improvement exhibited a significant quadratic response (*p* < 0.05, R^2^ = 0.821), and the estimated optimal inclusion level was 21.87 g kg^−1^.

A similar trend was observed for weight gain (WG), which increased from 41.78 ± 0.92 g in the control diet to 46.52 ± 0.55 g in the FLP20 treatment. WG also followed a significant quadratic pattern (*p* < 0.05, R^2^ = 0.8318), with an estimated optimum of 21.91 g kg^−1^ dietary inclusion.

Specific growth rate (SGR) improved correspondingly, reaching a maximum of 2.35 ± 0.01%/day in the FLP20 group, compared with 2.21 ± 0.02%/day in the control. This parameter also demonstrated a significant quadratic effect (*p* < 0.05, R^2^ = 0.8616), with the optimal inclusion level estimated at 21.83 g kg^−1^.

Feed conversion ratio (FCR) showed a numerical reduction from 1.09 ± 0.02 (control) to 1.05 ± 0.02 (FLP20), following a quadratic trend (R^2^ = 0.7095) with an estimated optimum of 20.64 g kg^−1^, although the differences were not statistically significant (*p* > 0.05).

Survival rate (SR) remained consistently high across all treatments, ranging from 91.67% to 96.67%, with no significant differences among groups (*p* > 0.05).

### 3.2. In Vitro Digestibility

The *in vitro* digestibility results are presented in Table 6. After the 8-week feeding trial, significant differences in intestinal carbohydrate digestibility were detected among treatments, with values ranging from 2.35 ± 0.05 to 2.91 ± 0.03 μmol maltose/g feed per unit of amylase activity. Fish fed the FLP20 diet exhibited the highest carbohydrate digestibility (2.91 ± 0.03), followed by the FLP40 (2.72 ± 0.05); both were significantly greater than that of the control group (FLP0; 2.35 ± 0.05) (*p* < 0.05). Protein digestibility showed a similar pattern, increasing significantly with FLP supplementation. The FLP20 treatment showed the highest protein digestibility (28.62 ± 1.10), which was significantly greater than that of the control (23.54 ± 0.13) (*p* < 0.05). Although the FLP5 and FLP40 groups also exhibited higher protein digestibility than the control, their values were not significantly different from those observed in the FLP20 group.

### 3.3. Digestive Enzyme Activity

The intestinal digestive enzyme activities of Nile tilapia fed diets containing different levels of fermented longan peel (FLP) for eight weeks are presented in Table 7. Amylase activity was significantly affected by dietary treatment (*p* < 0.05), increasing from 48.05 ± 2.22 µmol maltose/hr./mg protein in the control group to a maximum of 57.15 ± 1.88 μmol maltose h^−1^ mg^−1^ protein in the FLP20 group. Fish fed the FLP5, FLP10, and FLP40 diets showed intermediate amylase activities that were not significantly different from either the control or the FLP20 group. Lipase activity did not differ significantly among treatments (*p* > 0.05), with values ranging from 0.13 ± 0.01 to 0.15 ± 0.01 µmol p-nitrophenol/hr./mg protein, indicating that dietary FLP supplementation had no detectable effect on lipid digestion.

Protease activity varied significantly among treatments (*p* < 0.05). The highest activity was observed in the FLP20 group (3.24 ± 0.06 U/hr./mg protein), followed by the FLP40 group (3.06 ± 0.06); both values were significantly higher than that of the control group (2.77 ± 0.08 U h^−1^ mg^−1^ protein). The FLP10 group exhibited the lowest protease activity (2.58 ± 0.03 h^−1^ mg^−1^ protein) among treatments. Trypsin activity was also significantly influenced by dietary FLP supplementation (*p* < 0.05). Fish fed the FLP20 diet showed the highest trypsin activity (9.58 ± 0.06 µmol p-nitroaniline/hr./mg protein), whereas the other treatments showed lower but statistically similar values ranging from 8.30 to 8.48 μmol p-nitroaniline h^−1^ mg^−1^ protein.

### 3.4. Intestinal Microstructure

**Figure 2:**
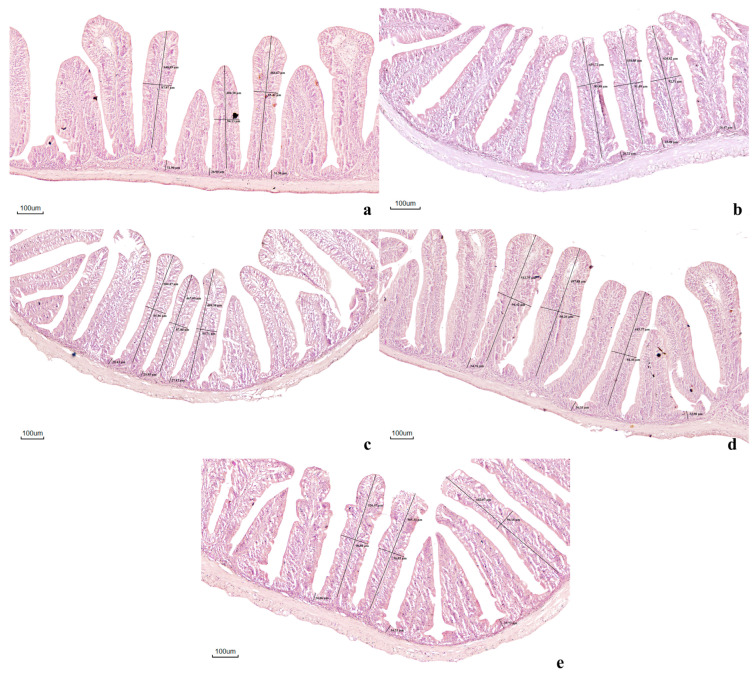
Histological representations of the H&E-stained intestinal sections of Nile tilapia. (**a**) Control group (FLP0), (**b**) FLP5: fermented longan peel at 5 g kg^−1^ diet, (**c**) FLP10: fermented longan peel at 10 g kg^−1^ diet, (**d**) FLP20: fermented longan peel at 20 g kg^−1^ diet, and (**e**) FLP40: fermented longan peel at 40 g kg^−1^ diet. Morphological features including villus height and crypt depth were observed across treatments. Scale bars represent 100 μm.

Histological examination revealed well-preserved intestinal structures across all treatments, characterized by normal villi and crypt organization (Figure 2 and Table 8). Fish fed FLP-supplemented diets showed improved gut morphology relative to the control group. Notably, the FLP10 and FLP20 treatments exhibited longer, more orderly villi, deeper crypts, and denser enterocyte arrangement. In contrast, the control (FLP0) and the highest inclusion level (FLP40) displayed shorter and less compact villi. These observations suggest that moderate FLP inclusion (10–20 g kg^−1^) promotes favorable intestinal structural development in Nile tilapia.

### 3.5. Innate Immune Response

#### 3.5.1. Mucosal Immune Response

Dietary supplementation with FLP did not produce significant changes in skin mucus lysozyme activity (MLA) or peroxidase activity (MPA) during the first 4 weeks of feeding (Table 9). However, after 8 weeks, both immune parameters increased markedly in fish receiving higher FLP inclusion levels (FLP20 and FLP40) compared with the control group (*p* < 0.05; Table 9). At week 4, MLA and MPA values remained comparable across all treatments (*p* > 0.05). By week 8, MLA reached its highest level in fish fed FLP40 (17.32 ± 0.62 μg mL^−1^), significantly surpassing the control group (12.03 ± 0.33 μg mL^−1^) (*p* < 0.05), whereas fish in the FLP10 and FLP20 groups showed intermediate but statistically similar responses. Likewise, although MPA did not vary among diets at week 4, a significant increase was recorded in the FLP20 group at week 8 (0.52 ± 0.02), more than doubling the activity observed in control fish (0.25 ± 0.02) (*p* < 0.05).

#### 3.5.2. Serum Immune Responses

Dietary supplementation with fermented longan peel (FLP) led to significant improvements in several serum innate immune parameters in Nile tilapia after both 4 and 8 weeks of feeding (*p* < 0.05; Table 10). After 4 weeks, serum lysozyme activity (SLA) was markedly higher in fish receiving the 40 g kg^−1^ FLP diet compared with the control group (*p* < 0.05), while the remaining treatments showed intermediate, non-significant differences. By week 8, SLA was significantly elevated in fish fed 20 and 40 g kg^−1^ FLP relative to the control, whereas other FLP levels produced moderate responses without statistical differences.

Serum peroxidase activity (SPA) also responded to FLP inclusion. At week 4, SPA was significantly increased in the 20 g kg^−1^ group compared with the control fish (*p* < 0.05). At week 8, however, only the 5 g kg^−1^ FLP group exhibited a significantly higher SPA value relative to the control group.

Alternative complement pathway activity (ACH50) showed a consistent enhancement, with the 20 g kg^−1^ FLP diet producing significantly higher ACH50 titres at both sampling points than their respective controls (*p* < 0.05). Other FLP treatments generally yielded intermediate responses, although the 5 g kg^−1^ group at week 4 was significantly lower than the 20 g kg^−1^ group.

Overall, FLP supplementation, especially at 20 g kg^−1^, substantially improved non-specific serum immune responses, demonstrating a clear immunostimulatory effect towards the end of the feeding trial.

### 3.6. Intestinal Growth, Immune, and Antioxidant-Related Gene Expression

**Figure 3:**
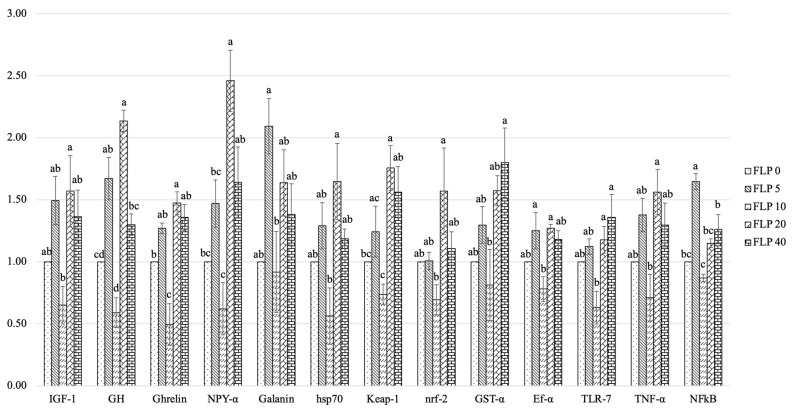
Relative transcript expression levels of growth, immune, and antioxidant-related genes in the intestine of Nile tilapia after 8 weeks feeding trial with fermented longan peel (FLP). B-actin was used as an internal reference gene. Data are presented as mean ± SEM. Different superscript letters indicate statistically significant differences (*p* < 0.05).

The influence of dietary fermented longan peel (FLP) on the transcription of genes associated with growth regulation, immune function, and antioxidant defense in the intestinal tissue of Nile tilapia is presented in Figure 3. Overall, fish receiving FLP-supplemented diets, particularly those fed 20 and 40 g kg^−1^, showed pronounced elevations in several target genes (*p* < 0.05). Marked increases were detected for *IGF-1*, *GH*, *Ghrelin*, *NPY-α*, *Galanin*, *hsp70*, *Keap-1*, *nrf-2*, *GST-α*, *Ef-α*, *TLR-7*, *TNF-α*, and *NFκB*, demonstrating broad enhancement of physiological pathways associated with growth, immune competence, and oxidative stress mitigation.

Growth-related markers such as *NPY-α* and Galanin exhibited the strongest responses, with transcript abundance in FLP 20 and FLP 40 groups approximately 1.5–2.1 times greater than that of the control, and significantly higher than responses observed in fish fed lower inclusion levels (FLP 5 and FLP 10). Antioxidant-related genes (*hsp70*, *Keap-1*, *nrf-2*, *GST-α*, and *Ef-α*) also showed clear upregulation in the FLP 20 and FLP 40 treatments, reflecting enhancements of roughly 1.2–1.7-fold over the control group, indicating improved cellular protective capacity. In contrast, fish receiving 5 or 10 g kg^−1^ FLP generally did not exhibit significant changes relative to unsupplemented fish.

Immune-regulatory genes, namely *TLR-7*, *TNF-α*, and *NFκB*, likewise increased by approximately 1.3–1.6-fold in the FLP 20 and FLP 40 groups, whereas expression levels in FLP 0, FLP 5, and FLP 10 remained statistically similar. Moderate, non-significant improvements were observed for certain genes (e.g., *IGF-1*, *GH*, *Ghrelin*) in fish fed 10 g kg^−1^ FLP, but these responses did not differ meaningfully from either the control or FLP 5 groups.

Notably, no substantial differences were detected between the FLP 20 and FLP 40 groups for most evaluated genes, suggesting that the transcriptional response tends to plateau beyond 20 g kg^−1^ dietary inclusion. The lowest gene expression levels consistently occurred in the control and FLP 5 treatments.

In summary, incorporating 20–40 g kg^−1^ FLP into Nile tilapia diets markedly stimulated intestinal gene expression related to growth regulation, innate immune function, and antioxidative defense, reaching increases of up to ~2.5-fold relative to the control, while lower supplementation levels produced limited or negligible effects.

## 4. Discussion

Sustainable aquaculture increasingly depends on innovative nutritional strategies that enhance fish growth, health, and resilience while supporting environmental and economic sustainability goals [65]. One promising approach is the valorization of agro-industrial by-products as functional feed additives, which contributes to waste reduction and promotes a circular bioeconomy. Among various strategies, fermentation of plant residues has gained growing attention because it can improve the nutritional quality, digestibility, and biological activity of feed materials. Fermented products often showed enhanced antioxidant and immunomodulatory properties due to the degradation of complex polysaccharides, reduction of antinutritional factors, and increased availability of bioactive compounds [66]. In the present study, dietary supplementation with fermented longan peel (FLP) significantly improved growth performance, digestive enzyme activity, immune responses, and the expression of growth-, immune-, and antioxidant-related genes in Nile tilapia (*Oreochromis niloticus*).

Growth performance is a key indicator of feed efficiency and overall physiological status in aquaculture species [67]. In the present study, fish receiving 20 g kg^−1^ of FLP showed significant improvements in growth performance and feed efficiency compared with the control group. Although research on fermented longan peel in fish nutrition remains limited, similar growth-promoting effects have been reported with other fermented agro-industrial by-products. For example, fermented corn cob improved growth performance and feed efficiency in Nile tilapia [22], while fermented sweet potato residue enhanced nutrient utilization and immune status in common carp (*Cyprinus carpio*) [68]. Comparable results have also been reported in European seabass (*Dicentrarchus labrax*) [69] fed solid-state fermented brewer’s spent grain; Nile tilapia fed fermented rice bran [70], fermented *Sargassum muticum* [21], fermented soybean meal [71], and fermented corn husk [25]. Fermentation can improve the nutritional value of plant ingredients by reducing fiber and antinutritional compounds while increasing the availability of nutrients and bioactive components [72]. These improvements may enhance nutrient digestibility and utilization, thereby supporting better growth performance [24]. Moreover, the presence of lactic acid bacteria, such as *Lactiplantibacillus plantarum*, may also contribute to the modulation of gut microbiota, fostering a balanced microbial community that supports nutrient absorption and intestinal health [73,74]. In the present study, enhanced digestive enzyme activities, particularly amylase, protease, and trypsin, were observed in fish fed the FLP20 diet. Similar enhancements in digestive physiology have been reported in fish fed fermented plant-based feed additives and phytogenic ingredients [75,76]. The fermentation process may increase the availability of nutrients and bioactive compounds that support digestive function and intestinal physiology [17,77].

Histological observations further supported these findings. Nile tilapia fed FLP-supplemented diets exhibited clear improvements in intestinal microstructure compared with the control group. Fish in the FLP10 and FLP20 groups displayed taller and more organized villi, deeper crypts, and well-aligned enterocytes, indicating improved intestinal health and absorptive capacity. Increased villus height expands the absorptive surface area of the intestine, facilitating more efficient nutrient uptake, while deeper crypts indicate active epithelial renewal and mucosal integrity [78]. The improved intestinal microstructure observed in the FLP-fed groups may be associated with the antioxidant properties of polyphenols and flavonoids present in fermented longan peel [14]. These compounds are known to help maintain epithelial integrity and protect intestinal tissues from oxidative stress [79,80]. Similar improvements in intestinal microstructure have been reported in African catfish (*Clarias gariepinus*) fed fermented spent coffee ground [81], spotted seabass (*Lateolabrax maculatus*) fed compound probiotics fermented soybean meal [71], and Nile tilapia (*Oreochromis niloticus*) fed fermented rice hulls [82] and fermented *Sargassum muticum* [21]. In the present study, the improved intestinal microstructure observed in the FLP10 and FLP20 groups is consistent with the enhanced digestive enzyme activities and growth performance.

Fish rely heavily on their skin mucus and serum innate immune components as the first line of defense against environmental and pathogenic challenges [83,84]. Dietary supplementation with functional feed additives has been reported to enhance immune responses and improve disease resistance in aquaculture species [85,86]. In the present study, dietary FLP supplementation significantly increased lysozyme, peroxidase, and alternative complement (ACH50) activities in both serum and skin mucus of Nile tilapia. These parameters play important roles in nonspecific immune defense mechanisms. Similar immunostimulatory effects have been reported with fermented feed additives, including fermented *Sargassum muticum* [21], fermented corn husk and corn cob [22,25], fermented soybean meal [71], and pomelo (*Citrus grandis*) peel and soybean meal co-fermented protein [87]. The improved immune responses observed in the present study may be associated with the polyphenol and flavonoid content of fermented longan peel (Table 1), as these compounds possess antioxidant and immunomodulatory properties [88]. Second, the fermentation process generates bioactive metabolites, such as lactic acid, peptides, and exopolysaccharides, which may serve as immunostimulants by activating macrophages, complement pathways, and pattern-recognition receptors [89,90,91]. Moreover, probiotic microorganisms like *Lactiplantibacillus plantarum* present in the fermented substrate may directly modulate gut-associated lymphoid tissue (GALT) and promote mucosal immune responses through improved microbial balance and short-chain fatty acid production [92,93]. Gene expression analysis provided further insight into the physiological mechanisms underlying the observed responses [94]. Dietary FLP supplementation significantly upregulated genes associated with growth regulation (*IGF-1*, *GH*, *Ghrelin*, *NPY-α*, and *Galanin*), immune responses (*TLR-7*, *TNF-α*, *NFκB*), and antioxidant defense (*hsp70*, *Keap-1*, *nrf-2*, *GST-α*, and *Ef-α*). It should also be noted that the transcriptional responses observed in the FLP10 group did not consistently follow a linear dose–response pattern. Such variations are commonly reported in nutritional gene expression studies, as gene regulation is influenced by complex physiological feedback mechanisms and individual biological variability. In the present study, the most consistent transcriptional activation occurred in the FLP20 group, which corresponded with the superior growth performance, digestive enzyme activity, and immune responses observed at the physiological level. The upregulation of GH and IGF-1 corresponds with the improved growth performance observed in FLP-fed fish, as these genes play key roles in somatic growth regulation and protein synthesis in fish [95,96,97]. Similar transcriptional responses have been reported in fish fed fermented plant-based feed additives, including fermented rice bran [70], fermented corn husk and corn cob [25], fermented *Sargassum muticum* [21], and fermented spent coffee ground [98]. Increased expression of immune-related genes such as *TLR-7*, *TNF-α*, and *NF-κB* indicates activation of innate immune signaling pathways [99,100], which is consistent with the elevated immune parameters observed in serum and mucus. Moreover, the upregulation of antioxidant-related genes including *nrf-2*, *Keap-1*, *GST-α*, and *hsp70* suggests enhanced antioxidant defense capacity and improved tolerance to oxidative stress [101].

An important contextual factor of this study is that Nile tilapia was cultured within a biofloc technology (BFT) system, which is characterized by complex microbial processes that contribute to nutrient recycling and water quality management. Biofloc systems stimulate heterotrophic bacterial growth through carbon supplementation and manipulation of the carbon-to-nitrogen ratio [102]. The microbial aggregates generated in BFT systems contain proteins, enzymes, vitamins, and immunologically active compounds that can contribute to fish nutrition and health [103,104]. Therefore, the physiological responses observed in this study likely reflect an interaction between dietary FLP supplementation and the biofloc culture environment. The polyphenols and flavonoids present in fermented longan peel may complement the nutritional and immunological benefits associated with biofloc systems, thereby contributing to improved digestion, immune responses, and antioxidant capacity. The absence of additional benefits at the highest FLP inclusion level (40 g kg^−1^) suggests that excessive inclusion of fermented plant materials may reduce dietary balance or limit nutrient utilization efficiency. Similar threshold responses have been reported for other phytogenic feed additives in aquaculture diets [24]. These findings indicate that moderate inclusion levels of FLP provide the most favorable physiological outcomes.

## 5. Conclusions

The present results demonstrate that dietary supplementation with fermented longan peel at an inclusion level of 20 g kg^−1^ can enhance growth performance, digestive function, intestinal microstructure, immune responses, and antioxidant defense in Nile tilapia cultured under biofloc conditions. These findings support the potential application of fermented longan peel as a sustainable phytogenic feed additive for improving fish health and productivity while contributing to the valorization of agricultural by-products in aquaculture systems.

## Figures and Tables

**Table 1:** Proximate analysis of fermented longan peel (FLP).

Parameter	FLP
Moisture (%)	8.2
Protein (%)	12.4
Fat (%)	1.19
Fiber (%)	48.3
Ash (%)	9.64

**Table 2:** Phenolic compounds of fermented longan peel (mg/100 g dry weight).

Compound	Longan Peel	Fermented Longan Peel (FLP)	Methods
Gallic acid	29.59	29.57	HPLC-PDA
Catechin	203.11	Nd.	HPLC-PDA
Epicatechin	161.34	Nd.	HPLC-PDA
Caffeic acid	Nd.	Nd.	HPLC-PDA
Epicatechin gallate	Nd.	Nd.	HPLC-PDA
p-Coumaric acid	Nd.	Nd.	HPLC-PDA
Naringin	105.70	Nd.	HPLC-PDA
Rosmarinic acid	59.35	11.07	HPLC-PDA
o-Coumaric acid	14.65	8.52	HPLC-PDA
Quercetin	Nd.	10.19	HPLC-PDA

Nd. = Not detected.

**Table 3:** Feed ingredients and proximate composition (g kg^−1^) of fermented longan peel (FLP).

	FLP 0	FLP 5	FLP 10	FLP 20	FLP 40
Fish meal	150	150	150	150	150
Corn meal	200	200	200	200	200
Soybean meal	390	390	390	390	390
Wheat flour	70	70	70	70	70
Rice bran	150	145	140	130	110
FLP	0	5	10	20	40
Binder	20	20	20	20	20
Soybean oil	2	2	2	2	2
Premix ^1^	10	10	10	10	10
Vitamin C ^2^	8	8	8	8	8
Proximate composition of the experimental diets (% of dry matter basis)					
Dry matter	94.18	93.99	94.19	94.18	93.79
Crude protein	31.39	32.01	31.32	31.56	31.23
Crude lipid	1.49	1.51	1.40	1.59	1.52
Ash	8.67	8.41	8.52	8.48	8.14
Fiber	4.32	4.67	4.75	5.34	5.93
GE (kcal/g) ^3^	4.03	4.04	4.02	4.03	4.01

^1^ Vitamin and trace mineral mix supplemented as follows (IU kg^−1^ or g kg^−1^ diet): retinyl acetate 1,085,000 IU; cholecalciferol 217,000 IU; D, L-a-tocopherol acetate 0.5 g; thiamin nitrate 0.5 g; pyridoxine hydrochloride 0.5 g; niacin 3 g; folic 0.05 g; cyanocobalamin 10 g; Ca pantothenate 1 g kg^−1^; inositol 0.5 g; zinc 1 g; copper 0.25 g; manganese 1.32 g; iodine 0.05 g; sodium 7.85 g; ^2^ Vitamin C 98% 5 g; ^3^ GE = gross energy.

**Table 4:** Primer sequences, amplicons, and associated information for quantitative real-time PCR of fermented longan peel.

Gene	Sequence	Accession Number	Reference
*Beta-actin*	F: CAGCAAGCAGGAGTACGATGAG R: TGTGTGGTGTGTGGTTGTTTTG	XM_003443127.4	[58]
*IGF-I: Insulin-growth factor 1*	F: GTCTGTGGAGAGCGAGGCTTT R: CACGTGACCGCCTTGCA	NM_001279503	[58]
*GH: Growth hormone*	F: TCGGTTGTGTGTTTGGGCGTCTC R: GTGCAGGTGCGTGACTCTGTTGA	XM_003442542	[58]
*Ghrelin*	F: GTGGTGCAAGTCAACCAGTG R: CATGGCTTGGCGACCAATTC	AB104859.1	[59]
*NPY-α F*	F: TCTCGCTCACTGCTGTCCC R: CAGAGGCGTGGTGTTCGTT	XM_003448854.5	[60]
*Galanin*	F: TGTTAGGGCCCCATGGACTA R: GAAGTCCTCCTCCTGGCCTA	XM_003453581.5	[59]
*HSP70 = Heat Shock Protein 70*	F: TTCAAGGTGATTTCAGACGGAG R: CTTCATCTTCACCAGGACCATG	XM_019357557.1	[61]
*keap1*	F: CTTCGCCATCATGAACGAGC R: CACCAACTCCATACCGCACT	XM_003447926.3	[61]
*Nrf2*	F: CTGCCGTAAACGCAAGATGG R: ATCCGTTGACTGCTGAAGGG	XM_003447296.4	[61]
*GST-a*	F: ACTGCACACTCATGGGAACA R: TTAAAAGCCAGCGGATTGAC	NM_001279635	[62]
*EF-α*	F: CTACAGCCAGGCTCGTTTCG R: CTTGTCACTGGTCTCCAGCA	AB075952	[63]
*TLR-7*	F: TCAGCAGGGTGAGAGCATAC R: ACATATCCCAGCCGTAGAGG	XM_005477981.1	[64]
*TNFɑ*	F: CCAGAAGCACTAAAGGCGAAGA R: CCTTGGCTTTGCTGCTGATC	NM_001279533.1	[58]
*nf-κB*	F: GAACATCAGACCGACGACCA R: TCTCCGCCAGTTTCTTCCA	XM_003457469.4	[61]

**Table 5:** Growth performance of utilization of Nile tilapia fed with different dietary levels of fermented longan peel.

	FLP 0	FLP 5	FLP 10	FLP 20	FLP 40
IW (g)	15.08 ± 0.02 ^a^	15.05 ± 0.03 ^a^	15.00 ± 0.00 ^a^	15.02 ± 0.02 ^a^	15.02 ± 0.02 ^a^
FW (g)					
4 weeks	30.72 ± 1.05 ^a^	32.53 ± 0.32 ^a^	30.95 ± 0.93 ^a^	31.97 ± 0.88 ^a^	29.9 ± 2.06 ^a^
8 weeks	56.87 ± 0.94 ^b^	58.19 ± 0.58 ^ab^	58.29 ± 1.34 ^ab^	61.53 ± 0.55 ^a^	57.63 ± 0.73 ^ab^
WG (g)					
4 weeks	15.63 ± 1.05 ^a^	17.48 ± 0.30 ^a^	15.95 ± 0.93 ^a^	16.95 ± 0.88 ^a^	14.88 ± 2.07 ^a^
8 weeks	41.78 ± 0.92 ^b^	43.14 ± 0.61 ^ab^	43.29 ± 1.34 ^ab^	46.52 ± 0.55 ^a^	42.61 ± 0.73 ^ab^
SGR (%/day)					
4 weeks	2.37 ± 0.11 ^a^	2.57 ± 0.03 ^a^	2.41 ± 0.10 ^a^	2.52 ± 0.09 ^a^	2.28 ± 0.24 ^a^
8 weeks	2.21 ± 0.02 ^b^	2.25 ± 0.02 ^ab^	2.26 ± 0.04 ^ab^	2.35 ± 0.01 ^a^	2.24 ± 0.02 ^b^
FCR					
4 weeks	0.72 ± 0.02 ^a^	0.66 ± 0.02 ^a^	0.66 ± 0.00 ^a^	0.64 ± 0.03 ^a^	0.68 ± 0.04 ^a^
8 weeks	1.09 ± 0.02 ^a^	1.10 ± 0.03 ^a^	1.06 ± 0.02 ^a^	1.05 ± 0.02 ^a^	1.10 ± 0.05 ^a^
SR (%)					
4 weeks	93.33 ± 1.67 ^a^	91.67 ± 3.33 ^a^	96.67 ± 3.33 ^a^	96.67 ± 1.67 ^a^	96.67 ± 1.67 ^a^
8 weeks	95.00 ± 2.89 ^a^	91.67 ± 3.33 ^a^	96.67 ± 3.33 ^a^	93.33 ± 3.33 ^a^	93.33 ± 6.67 ^a^

IW = initial fish weight, FW: final fish weight, WG: weight gain, SGR: specific fish growth rate^−1^, FCR: feed conversion ratio, SR: survival rate. Different letters in the same row indicate significant differences (*p* < 0.05).

**Table 6:** *In vitro* digestibility of carbohydrate (μmol maltose g feed^−1^ amylase activity^−1^) and protein (μmol DL-alanine equivalent g feed^−1^ trypsin activity^−1^) in intestine of Nile tilapia after eight weeks of feeding with diets containing different levels of fermented longan peel (FLP).

	FLP 0	FLP 5	FLP 10	FLP 20	FLP 40
Carbohydrate	2.35 ± 0.05 ^c^	2.69 ± 0.05 ^b^	2.39 ± 0.03 ^c^	2.91 ± 0.03 ^a^	2.72 ± 0.05 ^ab^
Protein	23.54 ± 0.13 ^c^	26.8 ± 0.26 ^ab^	25.49 ± 0.44 ^bc^	28.62 ± 1.10 ^a^	28.07 ± 0.72 ^ab^

Different letters in a row denote significant differences (*p* < 0.05) and means ± SE.

**Table 7:** Intestinal digestive enzymes activities of Nile Tilapia fed diets with different levels of fermented longan peel (FLP) for eight weeks. Different letters in the same row indicate significant differences (*p* < 0.05).

	FLP 0	FLP 5	FLP 10	FLP 20	FLP 40
Amylase activity	48.05 ± 2.22 ^b^	54.85 ± 2.52 ^ab^	51.98 ± 1.32 ^ab^	57.15 ± 1.88 ^a^	54.77 ± 1.35 ^ab^
Lipase activity	0.15 ± 0.01 ^a^	0.14 ± 0.00 ^a^	0.13 ± 0.01 ^a^	0.13 ± 0.01 ^a^	0.14 ± 0.01 ^a^
Protease activity	2.77 ± 0.08 ^bc^	2.73 ± 0.14 ^bc^	2.58 ± 0.03 ^c^	3.24 ± 0.06 ^a^	3.06 ± 0.06 ^ab^
Trypsin activity	8.31 ± 0.14 ^b^	8.48 ± 0.11 ^b^	8.30 ± 0.20 ^b^	9.58 ± 0.06 ^a^	8.46 ± 0.09 ^b^

Amylase activity (μmol maltose/hr./mg protein), Lipase activity (μmol p-nitrophenol/hr./mg protein), Protease activity (U/hr./mg protein), and Trypsin activity (μmol p-nitroaniline/hr./mg protein).

**Table 8:** Impact of dietary supplementation with fermented longan peel on intestinal microstructure.

	FLP 0	FLP 5	FLP 10	FLP 20	FLP 40
VH (μm)	445.40 ± 13.29 ^b^	485.80 ± 11.37 ^ab^	449.40 ± 25.36 ^b^	500.60 ± 3.50 ^ab^	523.60 ± 14.62 ^a^
VW (μm)	88.51 ± 0.57 ^b^	92.88 ± 0.63 ^ab^	90.81 ± 1.84 ^ab^	93.73 ± 0.65 ^a^	93.05 ± 1.01 ^ab^
CD (μm)	33.74 ± 0.92 ^a^	30.08 ± 3.15 ^a^	31.39 ± 2.85 ^a^	28.11 ± 1.28 ^a^	30.32 ± 0.79 ^a^
VH:CD	13.34 ± 0.68 ^b^	16.53 ± 1.36 ^ab^	14.5 ± 0.60 ^ab^	18.06 ± 1.06 ^a^	17.53 ± 0.12 ^a^

VH: villus height; VW: villus width; CD: crypt depth; VH:CD: villus height per crypt depth ratio. Different letters in the same row indicate significant differences (*p* < 0.05).

**Table 9:** Skin mucus lysozyme and peroxidase activities of Nile tilapia after 4 and 8 weeks feeding with experimental fermented longan peel (FLP).

		FLP 0	FLP 5	FLP 10	FLP 20	FLP 40
4 weeks	SMLA	12.47 ± 1.13 ^a^	11.27 ± 1.56 ^a^	9.99 ± 0.66 ^a^	11.38 ± 0.99 ^a^	11.72 ± 1.36 ^a^
	SMPA	0.29 ± 0.03 ^a^	0.25 ± 0.04 ^a^	0.32 ± 0.03 ^a^	0.38 ± 0.04 ^a^	0.33 ± 0.05 ^a^
8 weeks	SMLA	12.03 ± 0.33 ^b^	11.62 ± 1.29 ^b^	13.67 ± 1.39 ^ab^	13.29 ± 1.30 ^ab^	17.32 ± 0.62 ^a^
	SMPA	0.25 ± 0.02 ^b^	0.44 ± 0.07 ^ab^	0.32 ± 0.08 ^ab^	0.52 ± 0.02 ^a^	0.39 ± 0.04 ^ab^

SMLA: Skin mucus lysozyme activity (μg mL^−1^); SMPA: Skin mucus peroxidase activity (μg mL^−1^). Different letters in the same row indicate significant differences (*p* < 0.05).

**Table 10:** Serum lysozyme and peroxidase activities of Nile tilapia after 4 and 8 weeks feeding with experimental fermented longan peel (FLP).

		FLP 0	FLP 5	FLP 10	FLP 20	FLP 40
4 weeks	SLA	6.68 ± 0.65 ^b^	7.88 ± 0.2 ^ab^	7.69 ± 0.36 ^ab^	8.46 ± 0.33 ^ab^	9.15 ± 0.73 ^a^
	SPA	0.12 ± 0.04 ^b^	0.37 ± 0.07 ^ab^	0.33 ± 0.07 ^ab^	0.43 ± 0.04 ^a^	0.29 ± 0.10 ^ab^
	ACH50	282.60 ± 22.20 ^bc^	253.60 ± 44.57 ^c^	306.70 ± 49.20 ^ab^	446.10 ± 12.97 ^a^	423.10 ± 23.74 ^ab^
8 weeks	SLA	5.65 ± 0.21 ^b^	7.12 ± 0.39 ^ab^	6.97 ± 0.17 ^ab^	7.46 ± 0.43 ^a^	7.13 ± 0.22 ^a^
	SPA	0.12 ± 0.05 ^b^	0.49 ± 0.02 ^a^	0.35 ± 0.13 ^ab^	0.44 ± 0.02 ^ab^	0.18 ± 0.09 ^ab^
	ACH50	280.90 ± 27.92 ^b^	357.70 ± 15.59 ^ab^	300.90 ± 39.71 ^b^	491.50 ± 26.94 ^a^	354.00 ± 34.93 ^ab^

SLA: Serum lysozyme activity (μg mL^−1^); SPA: Serum peroxidase activity (μg mL^−1^); ACH50: Alternative complement activity. Different letters in the same row indicate significant differences (*p* < 0.05).

## Data Availability

The data that support the findings of this study are available from the corresponding author upon reasonable request.
